# Who?

**Published:** 1886-03

**Authors:** 


Who was it captured the A. M. A.,
And with it badly got away,
And now, for shame, says "*nix-for-stay?*"
Dave Yandell.

And who, when he had President been,
Had no more *use* for 't;—left it then;—
Now charges others with his sin?
Dave Yandell.

Who joined "ye festive bolters," when
They flew the track—disgruntled men!
And acted like a setting hen?
Dave Yandell.

Who, of the immaculate eight bereft,
The Congress work has done the "heft,"
And at St. Louis will *not* get left?
Shoemaker!

